# Integrating Water Flow, Locomotor Performance and Respiration of Chinese Sturgeon during Multiple Fatigue-Recovery Cycles

**DOI:** 10.1371/journal.pone.0094345

**Published:** 2014-04-08

**Authors:** Lu Cai, Lei Chen, David Johnson, Yong Gao, Prashant Mandal, Min Fang, Zhiying Tu, Yingping Huang

**Affiliations:** 1 Collaborative Innovation Center for Geo-Hazards and Eco-Environment in Three Gorges Area, Hubei Province, Yichang, PR China; 2 Institute of Chinese Sturgeon Research, China Three Gorges Project Corporation, Yichang, PR China; 3 Engineering Research Center of Eco-environment in Three Gorges Reservoir Region, Ministry of Education, China Three Gorges University, Yichang, PR China; 4 School of Natural Sciences and Mathematics, Ferrum College, Ferrum, Virginia, United States of America; Universitat de Barcelona, Spain

## Abstract

The objective of this study is to provide information on metabolic changes occurring in Chinese sturgeon (an ecologically important endangered fish) subjected to repeated cycles of fatigue and recovery and the effect on swimming capability. Fatigue-recovery cycles likely occur when fish are moving through the fishways of large dams and the results of this investigation are important for fishway design and conservation of wild Chinese sturgeon populations. A series of four stepped velocity tests were carried out successively in a Steffensen-type swimming respirometer and the effects of repeated fatigue-recovery on swimming capability and metabolism were measured. Significant results include: (1) critical swimming speed decreased from 4.34 bl/s to 2.98 bl/s; (2) active oxygen consumption (i.e. the difference between total oxygen consumption and routine oxygen consumption) decreased from 1175 mgO_2_/kg to 341 mgO_2_/kg and was the primary reason for the decrease in *U*_crit_; (3) excess post-exercise oxygen consumption decreased from 36 mgO_2_/kg to 22 mgO_2_/kg; (4) with repeated step tests, white muscle (anaerobic metabolism) began contributing to propulsion at lower swimming speeds. Therefore, Chinese sturgeon conserve energy by swimming efficiently and have high fatigue recovery capability. These results contribute to our understanding of the physiology of the Chinese sturgeon and support the conservation efforts of wild populations of this important species.

## Introduction

Water flow is commonly believed to be of an important environmental factor to fish. Natural selection favors locomotor and respiration strategies appropriate for an existing flow [1]. Chinese sturgeon (*Acipenser sinensis*, Gray 1834) has been listed as Class I Endangered Species by the Chinese Government [2] and is also listed as Critically Endangered (CR) in the IUCN Red List of Threatened Species (version 2013.1). The species is anadromous and currently occurs only in the middle and lower reaches of the Yangtze River and near-shore in the Yellow and East China Seas [3]. Although captive breeding of Chinese sturgeon is now feasible [4], [5], wild populations have declined significantly because of dam construction, overfishing and pollution [6]–[10]. Chinese sturgeon once migrated further than any other sturgeon, over 3,200 km in the Yangtze River. Restoration of the wild sturgeon populations is difficult due to long maturation time, infrequent spawning and low productivity [11]–[12]. Although the species has a life span of 50–60 years, the species breeds only three or four times and the hatchling survival rate is estimated at <1%.

Dam construction has blocked spawning migration [13] and the sturgeon populations have declined dramatically [10]. Augmenting wild populations by release of captive breed sturgeon is a great achievement for species conservation but not sufficient. Building fishways that allow migration is another important part of the effort to maintain wild populations [14], [15]. Data on swimming capability and the physiological response to exercise and fatigue provide important design criteria for fishways [16]–[19].

Publications on Chinese sturgeon have been more frequent in recent years [20] but have focused on biochemistry and habitat assessment. Research on swimming capability and excess post-exercise oxygen consumption, for one repetition, has been reported (e.g., [21]–[24]. However, fish typically undergo multiple fatigue-recovery cycles while moving through fishways of large dams [25] and data on the effects of repeated fatigue-recovery is necessary for the design of effective fishways. In this study, the effect of repeated fatigue-recovery cycles on swimming capability and metabolism were investigated using a stepped velocity test in a Steffensen-type swimming respirometer. The study documents the change in respiration and swimming capability of fish undergoing repeated fatigue-recovery cycles. These results contribute to our understanding of the physiology of the Chinese sturgeon and support the conservation efforts of wild populations of this important species.

## Materials and Methods

### Ethics Statement

This study was conducted in strict accordance with the laws governing animal experimentation in China. The protocol was approved by the China Three Gorges University. All efforts were made to minimize suffering.

### Test fish

Juvenile sturgeon were obtained from the institute of Chinese sturgeon research, China Three Gorges Project Corporation, in Yichang, China (30°56′ N; 111°15′ E) and were of the second filial generation of cultured Chinese sturgeon. The sturgeon (body length: 10.89±0.11 cm and body weight 8.38±0.27 g, mean ± S.E.) were maintained in cylindrical tanks (80 cm in diameter and 90 cm deep). They were fed to satiation each morning at 8:00 am with Tubificidae. Water temperature varied from 19.3°C to 20.8°C and dissolved oxygen (*DO*) maintained above 7.0 mg/L. Ammonia-N and nitrite-N were lower than 0.050 mg/L and 0.007 mg/L, respectively. Forty eight hours prior to the experiment, feeding was interrupted to prevent the elevated oxygen consumption rate associated with digestion [26].

### Respirometer

The sturgeon were tested in a sealed 14 L Steffensen-type swimming respirometer submerged in a 55.7 L tank (84.0 cm long ×39.9 cm wide ×17.0 cm deep) to maintain temperature. A schematic diagram of the respirometer was included in an earlier publication [27]. Respirometer components include a motor with speed controller and propeller and a rectangular swimming chamber (4.5 L, 35.5 cm long ×11.0 cm wide ×11.5 cm deep) with a Lucite multi-aperture rectifier at the entrance to maintain laminar flow and a wire grid at the exit. Normal respirometer operating assumptions were made: (1) swimming speed (*U*) is equal to water flow speed as measured with an acoustic Doppler velocity meter (Nortek AS, Oslo, Norway), and (2) oxygen consumption can be calculated from the change in dissolved oxygen, as measured by an oxygen electrode (Hach HQ30d, Loveland, USA).

### Protocol for stepped velocity test

Ten sturgeons were tested and the body length (bl), fork length, total length and body mass of each sturgeon were measured. Before testing, fish were acclimated overnight in the respirometer at a very low flow speed (0.3∼0.5 bl/s) and then allowed to swim at 0.5 bl/s from 7:00–8:00 am the next morning [12], [28]. When testing began, flow speed was increased by 0.5 bl/s every 20 min and dissolved oxygen was measured at 5 min intervals. When the sturgeon was exhausted (swimming ceased and the fish rested against the wire grid for 20 s), flow speed was adjusted to 0.5 bl/s during the 30 min recovery period [27]. This comprises a single stepped velocity test and each sturgeon tested was subjected to four tests in succession to simulate a repeated cycle of fatigue and recovery.

### Data collection and analyses

All experimental data was expressed as mean ± S.E. and was analyzed and fitted using Origin (Version 8.1). The level of significance was determined using ANOVA Fisher LSD. Fit to mathematical models was evaluated following the Akaike information criterion *AIC* [29], [30] using the model correcting for finite sample size and frequently applied in behavioral ecology [31];
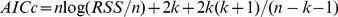
(1)where *RSS* is the residual sum of squares from the fitted model, *n* is sample size, and *k* is the total number of parameters. Its use helps prevent overfitting and offers a relative estimate of the information lost by a given model. The value of *AICc* is inversely related to the goodness of fit of a given model.

Based on data from the stepped velocity test, the critical swimming speed (*U*_crit_) of the test fish is calculated as described by Brett [32]. Oxygen consumption rate (*MO*_2_) is calculated using Equation 2; 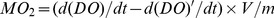
(2)where *V* (L) is the volume of the respirometer, *m* (kg) is the mass of the sturgeon, *d(DO)/dt* (mgO_2_/(L×h)), which is the slope of the linear regression of *DO* decreasing over time (*R* ^2^>0.99), is the rate of change in dissolved oxygen in the respirometer during the step test and *d(DO)'/dt* (mgO_2_/(L×h)) is the rate of change in dissolved oxygen in the control (respirometer with no fish). Routine oxygen consumption rate (*MO*_2, routine_) of each sturgeon is the *MO*_2_ at 0.5 bl/s (Cai et al., 2013b). Maximum oxygen consumption rate (*MO*_2, max_) of each sturgeon is the maximum value of *MO*_2_ during the stepped velocity test. The aerobic scope (*AS*) is the difference between *MO*_2, max_ and *MO*_2, routine_ and is related to the energy potentially available for swimming [33], [34].

During the swimming period, variation of *MO*_2_ with *U* was obtained by fitting the data to Equation 3 [35], [36]:
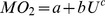
(3)where *a*, *b* and *c* are constants. Active oxygen consumption (*AOC*) is the difference between aerobic oxygen consumption and routine oxygen consumption during the period and was obtained by computing the area under the *MO*_2_ curve during the period. During the recovery period, variation of *MO*_2_ over time (*t*) was obtained by fitting the data to Equations 4 and 5 [37], [38]:
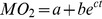
(4)




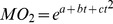
(5)where *a*, *b* and *c* are constants. Excess post-exercise oxygen consumption (*EPOC*) is the difference between aerobic oxygen consumption and routine oxygen consumption during recovery from exhaustion and was obtained by computing the area under the *MO*_2_ curve during the period. Assuming that *EPOC* is the oxygen consumed to recover from anaerobic metabolism, total oxygen consumption was obtained by adding *EPOC* to aerobic oxygen consumption [32]. Total oxygen consumption was calculated by the iterative method of Lee et al. [37] until *EPOC* and the area between the *MO*_2_ curve and the broken line (representing anaerobic metabolism) differed <0.5%. The broken line was then used to estimate *U* at the onset of anaerobic respiration (*U*a).

## Results

**Figure 1:**
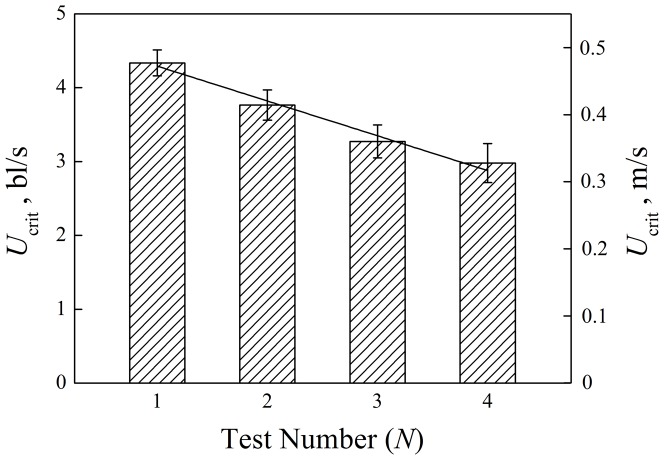
Variation of critical swimming speed (*U*_crit_, mean±S.E.) over four cycles of fatigue-recovery.

The *U*_crit_ of test sturgeon decreased with successive recoveries from fatigue (Fig. 1), yielding values of 4.34±0.18, 3.77±0.20, 3.27±0.22, 2.98±0.26 bl/s, respectively, over four cycles. The relationship between the *U*_crit_ and the test number (*N*) was linear and the decrease was significant:
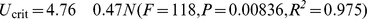



The *Δ_U_*_crit_ (*U*_crit, N+1_-*U*_crit, N_) values were 0.57, 0.50, 0.29 bl/s and the recovery ratios (*U*_crit, N+1_/*U*_crit, N_) were 86.9%, 86.7% and 91.1%.

Metabolic parameters were displayed in Table 1. Differences in *MO*_2, routine_ among the four tests were not significant. And differences in *MO*_2, max_ were also not significant; values for *MO*_2, routine_ were similar but *MO*_2, max_ and *AS* decreased with test number.

**Table 1:** Respiratory metabolic parameters*.

Test	*MO*_2, routine_	*MO*_2, max_	Mean *AS*	*AOC*	*EPOC*
	mgO_2_/(kg×h)	mgO_2_/(kg×h)	mgO_2_/(kg×h)	mgO_2_/kg	mgO_2_/kg
1	266.60±30.94	598.80±40.55	332.20	1,175.48	35.70
2	231.51±33.10	532.51±35.06	301.00	625.67	31.51
3	241.40±20.70	512.65±26.07	271.25	458.38	29.99
4	263.81±22.50	492.37±68.45	228.56	341.34	21.57

*Routine oxygen consumption rate (*MO*_2, routine_, mean ± S.E.) is the oxygen consumption rate (*MO*_2_) at 0.3∼0.5 BL/s; maximum oxygen consumption rate (*MO*_2, max_, mean ± S.E.) is the maximum value of *MO*_2_ during the stepped velocity test; the aerobic scope (*AS*) is the difference between *MO*_2, max_ and *MO*_2, routine_; active oxygen consumption (*AOC*) is the difference between aerobic oxygen consumption and routine oxygen consumption during swimming period; excess post-exercise oxygen consumption (*EPOC*) is the difference between aerobic oxygen consumption and routine oxygen consumption during recovery from exhaustion. Differences in *MO*_2, routine_ and *MO*_2, max_ not significant among successive tests: *MO*_2, routine_, *F* = 0.392, *P* = 0.759; *MO*_2, max_, *F* = 1.050, *P* = 0.390.

**Figure 2:**
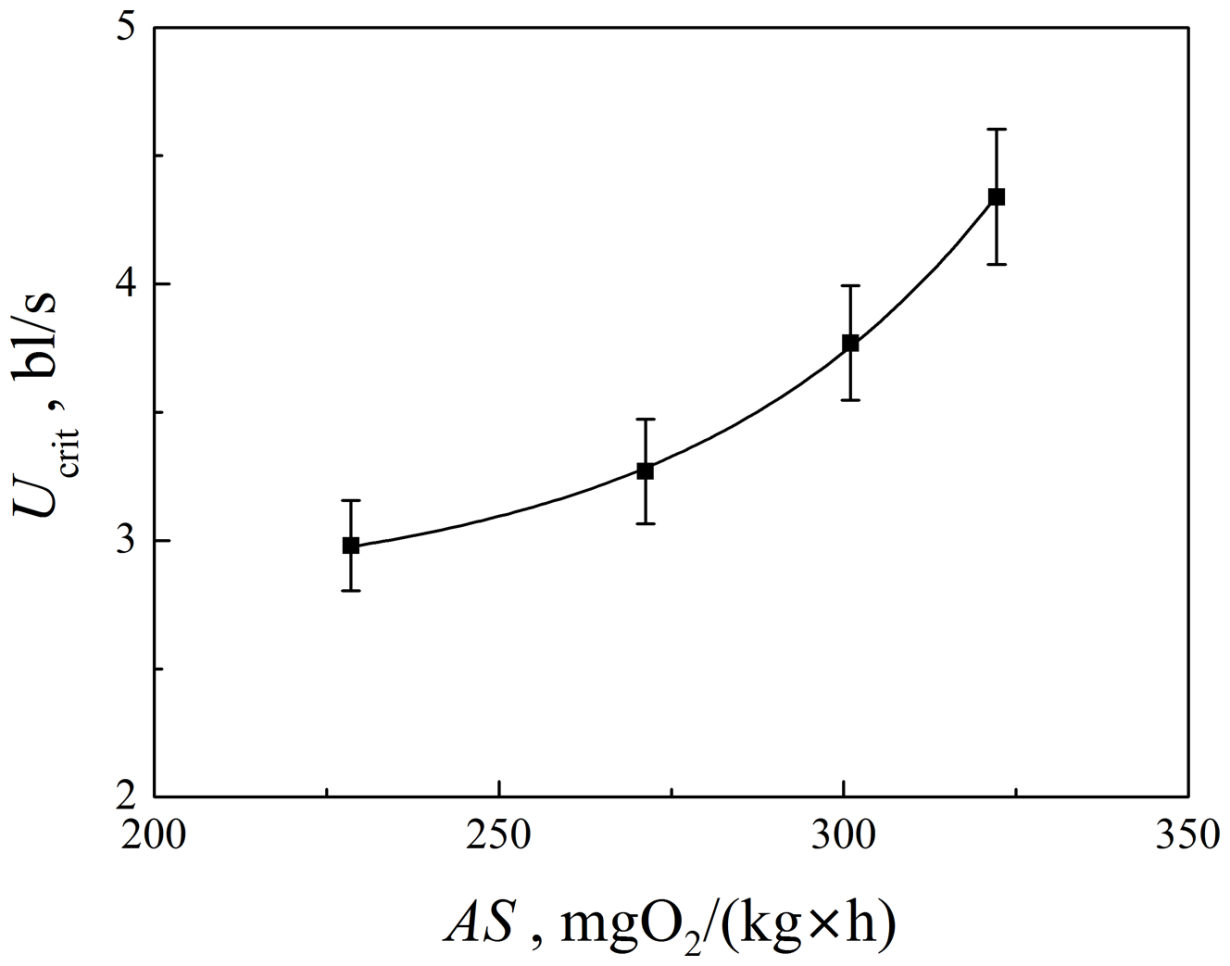
Variation of critical swimming speed (*U*_crit_, mean±S.E.) with aerobic scope (*AS*). The *AS* is the difference between maximum oxygen consumption rate and routine oxygen consumption rate.

As shown in Fig. 2, *U*_crit_ varied exponentially with *AS*:
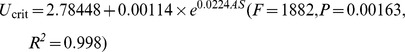



Data for the variation of *MO*_2_ with *U* was fitted to Equation 3 and the results are shown in Table 2. The speed coefficient (*c*), a measure of swimming efficiency, increased during tests 2 and 3 and then decreased in test 4.

**Table 2:** Relationship between oxygen consumption rate and swimming speed*.

Test	Equation	*F*	*P*	*R* ^2^
1	*MO*_2_ = 241.8+60.9*U* ^1.20^	1.6×10^6^	<0.00001	0.964
2	*MO*_2_ = 232.7+30.5*U* ^1.70^	0.8×10^6^	<0.00001	0.959
3	*MO*_2_ = 233.7+32.3*U* ^1.71^	6.3×10^6^	<0.00001	0.997
4	*MO*_2_ = 247.1+50.0*U* ^1.42^	8.1×10^6^	<0.00001	0.993

*Oxygen consumption rate, *MO*_2_, mgO2/(kg×h); swimming speed, *U*, bl/s.

**Figure 3:**
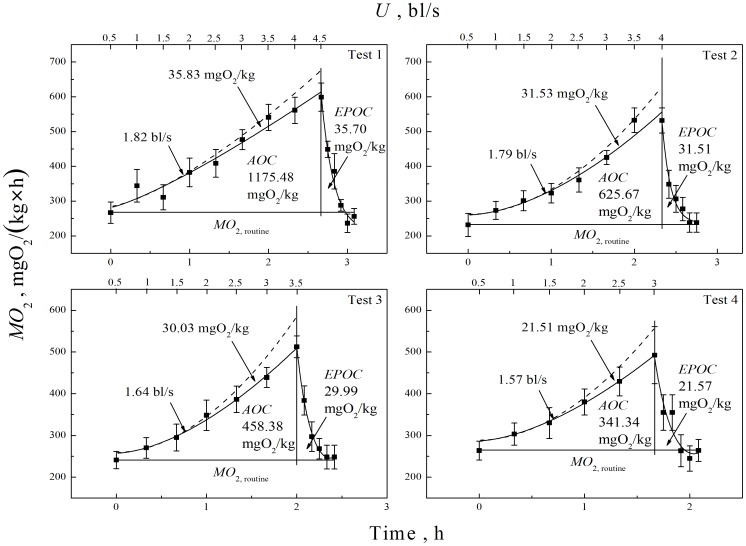
Variation of oxygen consumption rate (*MO*_2_, mean±S.E.) over time. Active oxygen consumption (*AOC*) is the difference between aerobic oxygen consumption and routine oxygen consumption during swimming period; excess post-exercise oxygen consumption (*EPOC*) is the difference between aerobic oxygen consumption and routine oxygen consumption during recovery from exhaustion.

Figure 3 illustrates the results of further analysis of the physiological changes that occurred during the four stepped velocity tests. The decreases in *EPOC* indicates that anaerobic capacity decreases with successive cycles of fatigue and recovery; the decrease in the last test was significant larger than in the second and third tests. The onset of anaerobic metabolism occurred at lower swimming speeds (absolute *U*_a_, 1.82, 1.79, 1.64, 1.57 bl/s, respectively, for tests 1∼4). However, because the decrease in *U*_crit_ was more pronounced, relative *U*_a_ increased with successive testing (41.94%, 47.48%, 50.15% and 52.68%).

During the recovery period, *MO*_2_ decreased rapidly over time; the relationship fitted to Equations 4 and 5 is shown in Table 3.

**Table 3:** Variation of oxygen consumption rate over time during the recovery period*.

Test	Equation	*F*	*P*	*R* ^2^
1	*MO*_2_ = 211.6+8.8×10^9^ *e* ^−6.35*t*^	0.5×10^6^	<0.00001	0.968
2	*MO*_2_ = 235.0+1.6×10^12^ *e* ^−9.61*t*^	1.1×10^6^	<0.00001	0.979
3	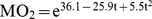	8.5×10^6^	<0.00001	0.998
4	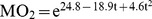	0.4×10^6^	<0.00001	0.837

*Oxygen consumption rate, *MO*_2_, mgO2/(kg×h); time, *t*, h.

## Discussion

### Critical swimming speed, *U*_crit_

Juvenile sturgeon, such as shortnose sturgeon *Acipenser brevirostrum* [39] and Amur sturgeon (*Acipenser schrenckii*) [27], generally display *U*_crit_ levels of 2 bl/s∼3 bl/s. However, the *U*_crit_ of Chinese sturgeon was found to be above that range, indicating that Chinese sturgeon have better swimming capability. In addition, there was a linear decrease (*R* ^2^ = 0.975) in *U*_crit_ of the Chinese sturgeon with repeated fatigue-recovery cycles (Fig. 1). However, the difference in *U*_crit_ declined with repeated testing (*Δ_U_*_crit_ 0.57, 0.50, 0.29 bl/s) and the recovery ratio increased from 86.9% to 91.1%. A better fit could have been obtained using a higher order equation but would have resulted in overfitting according to AIC analysis. The recovery ratio for Chinese sturgeon is comparable to sockeye salmon (*Oncorhynchus nerka*), with a recovery ratio of approximately 85% after a resting time of 1 hour [40]. Because exertion decreases *U*_crit_, the slot velocities of upstream fishways should be lower than those in downstream fishways.

### *MO*_2, routine_ and *MO*_2, max_

Differences in *MO*_2, routine_ over the four test cycles were small and erratic (Table 1), indicating little, if any, influence of fatigue on the routine metabolic rate. While differences in *MO*_2, max_ were not significant, the changes were more pronounced and decreased with each test cycle. This leads to a decrease in *AS* and is consistent with the observed decrease in the *U*_crit_. The relationship between *AS* and *U*_crit_ is shown in Fig. 2, indicating an exponential increase in *U*_crit_ with *AS*.

### Speed exponent, *c*

The exponent *c* (Equation 3) is inversely related to swimming efficiency [34], [38]. In the first test, *c* was 1.20, similar to those of the white sturgeon, lake sturgeon and Amur sturgeon [27], [41]; not surprising as the four *Acipenser* species are closely related [42]. In the three successive tests, the values of *c* did not change consistently (*c* = 1.70, 1.71, 1.42, respectively, for Tests 2, 3, and 4). There are numerous reports on the effect of fish morphology on swimming efficiency [43], [44]. In this investigation morphology was consistent among the tests and results indicate that repeated fatigue-recovery cycles affect physiological function. It has been reported that different fish use different swimming strategies [45], [46], but it remains unclear as to whether the sturgeon are adjusting swimming strategy in response to repeated fatigue-recovery cycles. Swimming efficiency was highest in Test 1, lower in Tests 2 and 3, with some recovery displayed in Test 4.

### Excess post-exercise oxygen consumption, EPOC

During fatigue recovery the variation of oxygen consumption rate with time is usually described by Equation 4 [27], [37]. The data fit for Test 1 and Test 2 was good (Table 3) but data for Test 3 and Test 4 could not be fit to Equation 4 because the fit did not converge. After fitting the data to several other functions Equation 5 was selected, based on fit and *AIC* analysis, to estimate the onset of anaerobic respiration and its contribution to *U*_crit_.

Information on *EPOC* is helpful for establishing design criteria for the resting pools of fishways [27], [47]. The *EPOC* is inversely related to the recovery capability from exhaustion [37]. The *EPOC* in the first test was 35.7 mgO_2_/kg, much lower than for salmon but similar to Amur sturgeon [27], [37], indicating high recovery capability for Chinese sturgeon. Although *EPOC* decreased with repeated recovery from fatigue, it does not necessarily follow that the recovery capability increased because *U*_crit_ and *AS* also decreased and *AOC* decreased by 70% from Test 1 to Test 4 (Table 1).

The *EPOC* is directly related to anaerobic respiration [37] and the decrease in *EPOC* indicates that repeated recovery from fatigue lowers capacity for anaerobic metabolism. With each fatigue-recovery cycle anaerobic respiration begins at a lower swimming speed but contributes less to achieving *U*_crit_. However, it can be seen in Fig. 3 and Table 1 that oxygen consumption is primarily from aerobic respiration and the decrease in aerobic respiration is the primary cause for the decrease in *U*_crit_. It was reported that salmon pass fishways using primarily aerobic respiration [25], consistent with our results.

### Swimming speed at onset of anaerobic respiration, *U*_a_

Aerobic metabolism with red muscle fiber provides propulsion in general, but white muscle fiber contributes to achieving high speeds [36], [48], [49]. It was reported that anaerobic swimming in cyprinids begins to augment propulsion when swimming speed reaches 30∼50% *U*_crit_ [50]. However, aerobic power is different in different fish species [37], [50], [51]. In this study, the relative *U*_a_ was between 41.94% and 52.68% *U*_crit_ (absolute *U*_a_, 1.82, 1.79, 1.64, 1.57 bl/s, respectively, for tests 1∼4) similar to the higher range of cyprinids. So, with repeated step tests, white muscle (anaerobic metabolism) began contributing to propulsion at lower absolute swimming speeds.

## Conclusions

Repeated cycles of fatigue and recovery affect the swimming capability of Chinese sturgeon; *U*_crit_, *AOC* and absolute *U*_a_ decrease with decreasing swimming capacity. These results contribute to our understanding of the physiology of the Chinese sturgeon and support the conservation efforts of wild populations of this important species.
